# Di-*n*-butyl­bis­(*N*-cyclo­hexyl-*N*-ethyl­dithio­carbamato-κ^2^ *S*,*S*′)tin(IV)

**DOI:** 10.1107/S1600536810027303

**Published:** 2010-07-17

**Authors:** Normah Awang, Ibrahim Baba, Bohari M. Yamin, Seik Weng Ng

**Affiliations:** a School of Chemical Sciences and Food Technology, Faculty of Science and Technology, Universiti Kebangbaan Malaysia, 43600 Bangi, Malaysia; b Department of Chemistry, University of Malaya, 50603 Kuala Lumpur, Malaysia

## Abstract

The Sn^IV^ atom in the title compound, [Sn(C_4_H_9_)_2_(C_9_H_16_NS_2_)_2_], is chelated by the two dithio­carbamate ions in a six-coordinate skew-trapezoidal-bipyramidal geometry. The two butyl groups are disordered over two positions in a 1:1 ratio.

## Related literature

For a discussion on six-coordinate, skew-trapezoidal-bipyram­idal diorganotin(IV) bis­(chelates), see: Ng *et al.* (1987 ▶).
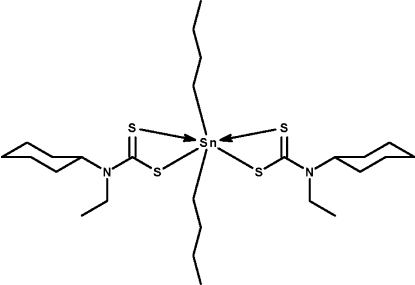


## Experimental

### 

#### Crystal data

[Sn(C_4_H_9_)_2_(C_9_H_16_NS_2_)_2_]
*M* *_r_* = 637.67
Triclinic, 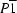

*a* = 10.2809 (4) Å
*b* = 12.5462 (4) Å
*c* = 13.3823 (5) Å
α = 103.103 (1)°
β = 108.125 (1)°
γ = 90.655 (1)°
*V* = 1591.5 (1) Å^3^
*Z* = 2
Mo *K*α radiation
μ = 1.08 mm^−1^
*T* = 293 K
0.35 × 0.25 × 0.25 mm

#### Data collection

Bruker SMART CCD diffractometer
Absorption correction: multi-scan (*SADABS*; Sheldrick, 1996 ▶) *T*_min_ = 0.703, *T*_max_ = 0.774
20772 measured reflections
7294 independent reflections
6264 reflections with *I* > 2σ(*I*)
*R*_int_ = 0.024

#### Refinement

*R*[*F* ^2^ > 2σ(*F* ^2^)] = 0.045
*wR*(*F* ^2^) = 0.125
*S* = 1.03
7294 reflections
316 parameters
56 restraints
H-atom parameters constrained
Δρ_max_ = 1.65 e Å^−3^
Δρ_min_ = −0.45 e Å^−3^

### 

Data collection: *SMART* (Bruker, 2002 ▶); cell refinement: *SAINT* (Bruker, 2002 ▶); data reduction: *SAINT*; program(s) used to solve structure: *SHELXS97* (Sheldrick, 2008 ▶); program(s) used to refine structure: *SHELXL97* (Sheldrick, 2008 ▶); molecular graphics: *X-SEED* (Barbour, 2001 ▶); software used to prepare material for publication: *publCIF* (Westrip, 2010 ▶).

## Supplementary Material

Crystal structure: contains datablocks global, I. DOI: 10.1107/S1600536810027303/zs2050sup1.cif

Structure factors: contains datablocks I. DOI: 10.1107/S1600536810027303/zs2050Isup2.hkl

Additional supplementary materials:  crystallographic information; 3D view; checkCIF report

## Figures and Tables

**Table:** 

Sn1—C1	2.128 (4)
Sn1—C5	2.134 (4)
Sn1—S3	2.5292 (9)
Sn1—S1	2.5425 (9)
Sn1—S4	2.8927 (9)
Sn1—S2	2.9257 (10)

**Table:** 

C1—Sn1—C5	145.4 (2)

**Fig. 1.**
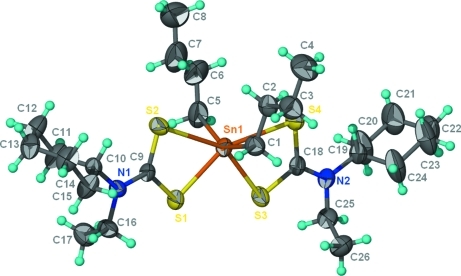
Thermal ellipsoid plot (Barbour, 2001) of (I) at the 50% probability level. The disorder in the butyl chains is not shown.

## supplementary crystallographic information

### Comment

Diorganotin(IV) bis(chelates) generally adopt six-coordinate geometries at tin, a geometry for which the carbon–tin–carbon bond angle is about 135° (Ng *et al.*, 1987). The dithiocarbamate ion is an example of a chelating ligand that imposes such a geometry on the tin atom in diorganotin(IV) compounds. The angle at tin is generally unafffected by the size of the organic group bonded to the metal atom or the steric size of the dithiocarbamate ion. In the title compound (I) (Scheme I, Fig. 1), this angle is 145.4 (2)° and the angle formed by the formally neutral sulfur atom is opened up to 144.13 (3)°.

### Experimental

Cabon disulfide (30 mmol) was dropped into an ethanol solution (100 ml) of *N*-cyclohexyl-*N*-ethylamine (30 mmol). The solution was kept at 273 K for an hour. Dibutyltin dichloride (15 mmol) dissolved in ethanol (100 ml) was added to give a white precipitate. This was collected and recrystallized from a chloroform/ethanol (1/1) mixture.

### Refinement

The carbon atoms of the butyl chains (except for the carbon atoms connected to the tin atom) show large thermal ellipsoids. The disorder could not be refined, and was assumed to be a 1:1 disorder. The 1,2-related carbon-carbon distances were restrained to 1.54±0.01 Å and the 1,3-related ones to 2.51±0.01 Å. The temperature factors of the primed atoms were set to those of the unprimed ones; the anisotropic temperature factors of the disordered atoms were restrained to be nearly isotropic. Carbon-bound H-atoms were placed in calculated positions (C—H = 0.93 to 0.97 Å) and were included in the refinement in the riding model approximation, with *U*_iso_(H) set to 1.2 to 1.5*U*_eq_(C). The final difference Fourier map had a peak in the vicinity of Sn1.

### Crystal data

**Table:**

[Sn(C_4_H_9_)_2_(C_9_H_16_NS_2_)_2_]	*Z* = 2
*M**_r_* = 637.67	*F*(000) = 668
Triclinic, *P*1	*D*_x_ = 1.331 Mg m^−^^3^
Hall symbol: -P 1	Mo *K*α radiation, λ = 0.71073 Å
*a* = 10.2809 (4) Å	Cell parameters from 7406 reflections
*b* = 12.5462 (4) Å	θ = 2.6–26.6°
*c* = 13.3823 (5) Å	µ = 1.08 mm^−^^1^
α = 103.103 (1)°	*T* = 293 K
β = 108.125 (1)°	Block, colorless
γ = 90.655 (1)°	0.35 × 0.25 × 0.25 mm
*V* = 1591.5 (1) Å^3^	

### Data collection

**Table:**

Bruker SMART CCD diffractometer	7294 independent reflections
Radiation source: fine-focus sealed tube	6264 reflections with *I* > 2σ(*I*)
graphite	*R*_int_ = 0.024
ω scans	θ_max_ = 27.5°, θ_min_ = 1.7°
Absorption correction: multi-scan (*SADABS*; Sheldrick, 1996)	*h* = −13→13
*T*_min_ = 0.703, *T*_max_ = 0.774	*k* = −16→16
20772 measured reflections	*l* = −17→17

### Refinement

**Table:**

Refinement on *F*^2^	Primary atom site location: structure-invariant direct methods
Least-squares matrix: full	Secondary atom site location: difference Fourier map
*R*[*F*^2^ > 2σ(*F*^2^)] = 0.045	Hydrogen site location: inferred from neighbouring sites
*wR*(*F*^2^) = 0.125	H-atom parameters constrained
*S* = 1.03	*w* = 1/[σ^2^(*F*_o_^2^) + (0.0681*P*)^2^ + 0.9862*P*] where *P* = (*F*_o_^2^ + 2*F*_c_^2^)/3
7294 reflections	(Δ/σ)_max_ = 0.001
316 parameters	Δρ_max_ = 1.65 e Å^−^^3^
56 restraints	Δρ_min_ = −0.44 e Å^−^^3^

### Fractional atomic coordinates and isotropic or equivalent isotropic displacement parameters (Å^2^)

**Table:**

	*x*	*y*	*z*	*U*_iso_*/*U*_eq_	Occ. (<1)
Sn1	0.42811 (2)	0.309809 (17)	0.227305 (19)	0.05422 (10)	
S1	0.36710 (10)	0.17378 (7)	0.04359 (7)	0.0581 (2)	
S2	0.30272 (14)	0.09498 (9)	0.21583 (9)	0.0814 (3)	
S3	0.52115 (11)	0.43790 (7)	0.13954 (7)	0.0623 (2)	
S4	0.54453 (12)	0.52211 (7)	0.36982 (8)	0.0670 (3)	
N1	0.2738 (3)	−0.0314 (2)	0.0204 (2)	0.0509 (6)	
N2	0.6358 (3)	0.6359 (2)	0.2562 (2)	0.0563 (7)	
C1	0.6110 (4)	0.2555 (3)	0.3212 (3)	0.0633 (9)	
H1A	0.6136	0.1780	0.2907	0.076*	0.50
H1B	0.6895	0.2947	0.3158	0.076*	0.50
H1C	0.6239	0.1835	0.2820	0.076*	0.50
H1D	0.6883	0.3052	0.3286	0.076*	0.50
C2	0.625 (3)	0.2722 (19)	0.4410 (9)	0.065 (3)	0.50
H2A	0.6211	0.3493	0.4723	0.078*	0.50
H2B	0.5490	0.2309	0.4478	0.078*	0.50
C3	0.761 (4)	0.234 (3)	0.5023 (15)	0.082 (4)	0.50
H3A	0.8365	0.2748	0.4950	0.098*	0.50
H3B	0.7641	0.1567	0.4716	0.098*	0.50
C4	0.775 (7)	0.252 (5)	0.6223 (16)	0.114 (5)	0.50
H4A	0.8621	0.2313	0.6607	0.171*	0.50
H4B	0.7026	0.2081	0.6296	0.171*	0.50
H4C	0.7677	0.3282	0.6518	0.171*	0.50
C2'	0.613 (3)	0.249 (2)	0.4344 (9)	0.065 (3)	0.50
H2'A	0.5650	0.3074	0.4634	0.078*	0.50
H2'B	0.5664	0.1792	0.4298	0.078*	0.50
C3'	0.761 (4)	0.258 (3)	0.5103 (14)	0.082 (4)	0.50
H3'A	0.8051	0.3299	0.5207	0.098*	0.50
H3'B	0.8118	0.2033	0.4783	0.098*	0.50
C4'	0.762 (7)	0.240 (5)	0.6199 (18)	0.114 (5)	0.50
H4'A	0.8551	0.2420	0.6658	0.171*	0.50
H4'B	0.7152	0.1691	0.6092	0.171*	0.50
H4'C	0.7160	0.2961	0.6534	0.171*	0.50
C5	0.2343 (4)	0.3697 (4)	0.2261 (4)	0.0931 (15)	
H5A	0.2353	0.4423	0.2123	0.112*	0.50
H5B	0.1650	0.3230	0.1641	0.112*	0.50
H5C	0.2268	0.4386	0.2042	0.112*	0.50
H5D	0.1607	0.3172	0.1745	0.112*	0.50
C6	0.1859 (17)	0.379 (3)	0.3209 (12)	0.121 (4)	0.50
H6A	0.2509	0.4268	0.3846	0.146*	0.50
H6B	0.1785	0.3068	0.3354	0.146*	0.50
C7	0.0420 (10)	0.4260 (15)	0.2955 (10)	0.135 (4)	0.50
H7A	0.0491	0.4974	0.2802	0.162*	0.50
H7B	−0.0233	0.3774	0.2323	0.162*	0.50
C8	−0.0064 (13)	0.4363 (14)	0.3911 (11)	0.138 (4)	0.50
H8A	−0.0922	0.4689	0.3778	0.207*	0.50
H8B	0.0606	0.4819	0.4540	0.207*	0.50
H8C	−0.0186	0.3648	0.4031	0.207*	0.50
C6'	0.2218 (15)	0.387 (3)	0.3376 (10)	0.121 (4)	0.50
H6'A	0.3050	0.4275	0.3904	0.146*	0.50
H6'B	0.2134	0.3159	0.3536	0.146*	0.50
C7'	0.0965 (11)	0.4501 (14)	0.3491 (13)	0.135 (4)	0.50
H7'A	0.1124	0.4850	0.4249	0.162*	0.50
H7'B	0.0865	0.5073	0.3098	0.162*	0.50
C8'	−0.0339 (12)	0.3750 (14)	0.3060 (13)	0.138 (4)	0.50
H8'A	−0.1101	0.4173	0.3098	0.207*	0.50
H8'B	−0.0272	0.3220	0.3487	0.207*	0.50
H8'C	−0.0475	0.3376	0.2320	0.207*	0.50
C9	0.3098 (3)	0.0674 (3)	0.0880 (3)	0.0513 (7)	
C10	0.2289 (4)	−0.1252 (3)	0.0557 (3)	0.0516 (7)	
H10	0.2508	−0.1009	0.1345	0.062*	
C11	0.0772 (4)	−0.1549 (3)	0.0106 (4)	0.0732 (11)	
H11A	0.0505	−0.1835	−0.0673	0.088*	
H11B	0.0294	−0.0899	0.0251	0.088*	
C12	0.0375 (4)	−0.2408 (4)	0.0621 (5)	0.0866 (14)	
H12A	0.0563	−0.2092	0.1389	0.104*	
H12B	−0.0604	−0.2620	0.0299	0.104*	
C13	0.1141 (4)	−0.3410 (3)	0.0475 (4)	0.0741 (11)	
H13A	0.0918	−0.3907	0.0869	0.089*	
H13B	0.0854	−0.3787	−0.0287	0.089*	
C14	0.2659 (4)	−0.3117 (3)	0.0872 (4)	0.0748 (11)	
H14A	0.3121	−0.3772	0.0710	0.090*	
H14B	0.2964	−0.2834	0.1653	0.090*	
C15	0.3059 (4)	−0.2258 (3)	0.0350 (4)	0.0719 (11)	
H15A	0.4041	−0.2055	0.0653	0.086*	
H15B	0.2835	−0.2560	−0.0423	0.086*	
C16	0.2768 (4)	−0.0508 (3)	−0.0916 (3)	0.0628 (9)	
H16A	0.2857	−0.1282	−0.1176	0.075*	
H16B	0.3576	−0.0099	−0.0913	0.075*	
C17	0.1515 (5)	−0.0186 (5)	−0.1699 (4)	0.0913 (14)	
H17A	0.1607	−0.0339	−0.2409	0.137*	
H17B	0.1432	0.0585	−0.1463	0.137*	
H17C	0.0709	−0.0599	−0.1722	0.137*	
C18	0.5741 (3)	0.5428 (3)	0.2578 (3)	0.0504 (7)	
C19	0.6807 (4)	0.7283 (3)	0.3531 (3)	0.0683 (10)	
H19	0.6562	0.7044	0.4102	0.082*	
C20	0.6089 (6)	0.8281 (4)	0.3381 (6)	0.118 (2)	
H20A	0.5104	0.8096	0.3140	0.142*	
H20B	0.6312	0.8554	0.2827	0.142*	
C21	0.6511 (6)	0.9177 (5)	0.4444 (6)	0.127 (2)	
H21A	0.6080	0.9839	0.4314	0.153*	
H21B	0.6187	0.8935	0.4971	0.153*	
C22	0.8011 (6)	0.9428 (4)	0.4886 (5)	0.1024 (17)	
H22A	0.8315	0.9774	0.4408	0.123*	
H22B	0.8243	0.9946	0.5587	0.123*	
C23	0.8737 (7)	0.8449 (5)	0.5006 (6)	0.129 (3)	
H23A	0.8528	0.8158	0.5557	0.154*	
H23B	0.9719	0.8648	0.5248	0.154*	
C24	0.8344 (5)	0.7562 (4)	0.3952 (5)	0.107 (2)	
H24A	0.8637	0.7819	0.3418	0.128*	
H24B	0.8804	0.6910	0.4075	0.128*	
C25	0.6604 (4)	0.6495 (3)	0.1565 (3)	0.0649 (9)	
H25A	0.6685	0.7273	0.1594	0.078*	
H25B	0.5809	0.6160	0.0948	0.078*	
C26	0.7854 (5)	0.6009 (4)	0.1381 (4)	0.0879 (13)	
H26A	0.7946	0.6142	0.0727	0.132*	
H26B	0.7768	0.5231	0.1317	0.132*	
H26C	0.8651	0.6340	0.1982	0.132*	

### Atomic displacement parameters (Å^2^)

**Table:**

	*U*^11^	*U*^22^	*U*^33^	*U*^12^	*U*^13^	*U*^23^
Sn1	0.05327 (15)	0.04494 (14)	0.06159 (16)	−0.00580 (9)	0.02086 (11)	0.00446 (10)
S1	0.0651 (5)	0.0473 (4)	0.0591 (5)	−0.0115 (4)	0.0193 (4)	0.0092 (4)
S2	0.1208 (9)	0.0606 (6)	0.0617 (6)	−0.0339 (6)	0.0472 (6)	−0.0101 (4)
S3	0.0791 (6)	0.0469 (4)	0.0528 (5)	−0.0110 (4)	0.0154 (4)	0.0054 (3)
S4	0.0905 (7)	0.0472 (4)	0.0623 (5)	−0.0179 (4)	0.0334 (5)	0.0003 (4)
N1	0.0617 (16)	0.0433 (13)	0.0463 (14)	−0.0040 (11)	0.0205 (12)	0.0045 (11)
N2	0.0621 (16)	0.0435 (14)	0.0628 (17)	−0.0069 (12)	0.0213 (13)	0.0106 (12)
C1	0.068 (2)	0.059 (2)	0.062 (2)	0.0050 (17)	0.0214 (17)	0.0114 (16)
C2	0.077 (4)	0.043 (8)	0.071 (3)	−0.001 (5)	0.024 (3)	0.007 (3)
C3	0.092 (3)	0.072 (11)	0.079 (4)	0.009 (7)	0.026 (3)	0.015 (5)
C4	0.135 (10)	0.124 (10)	0.074 (3)	0.007 (8)	0.015 (4)	0.036 (4)
C2'	0.077 (4)	0.043 (8)	0.071 (3)	−0.001 (5)	0.024 (3)	0.007 (3)
C3'	0.092 (3)	0.072 (11)	0.079 (4)	0.009 (7)	0.026 (3)	0.015 (5)
C4'	0.135 (10)	0.124 (10)	0.074 (3)	0.007 (8)	0.015 (4)	0.036 (4)
C5	0.061 (2)	0.086 (3)	0.114 (4)	−0.004 (2)	0.025 (2)	−0.006 (3)
C6	0.078 (8)	0.148 (7)	0.139 (7)	−0.012 (9)	0.059 (6)	0.002 (7)
C7	0.061 (7)	0.148 (10)	0.171 (13)	−0.010 (7)	0.044 (7)	−0.021 (9)
C8	0.107 (7)	0.180 (12)	0.127 (9)	0.003 (7)	0.056 (7)	0.010 (7)
C6'	0.078 (8)	0.148 (7)	0.139 (7)	−0.012 (9)	0.059 (6)	0.002 (7)
C7'	0.061 (7)	0.148 (10)	0.171 (13)	−0.010 (7)	0.044 (7)	−0.021 (9)
C8'	0.107 (7)	0.180 (12)	0.127 (9)	0.003 (7)	0.056 (7)	0.010 (7)
C9	0.0493 (16)	0.0474 (16)	0.0535 (17)	−0.0075 (13)	0.0177 (14)	0.0039 (13)
C10	0.0616 (19)	0.0418 (15)	0.0483 (16)	−0.0053 (13)	0.0161 (14)	0.0077 (12)
C11	0.054 (2)	0.070 (2)	0.113 (3)	0.0130 (17)	0.033 (2)	0.047 (2)
C12	0.057 (2)	0.084 (3)	0.136 (4)	0.006 (2)	0.035 (3)	0.058 (3)
C13	0.076 (3)	0.053 (2)	0.090 (3)	−0.0098 (18)	0.018 (2)	0.0249 (19)
C14	0.067 (2)	0.060 (2)	0.105 (3)	0.0150 (18)	0.025 (2)	0.038 (2)
C15	0.056 (2)	0.062 (2)	0.109 (3)	0.0129 (17)	0.033 (2)	0.036 (2)
C16	0.087 (3)	0.0499 (18)	0.0525 (19)	−0.0011 (17)	0.0286 (18)	0.0061 (14)
C17	0.100 (4)	0.104 (4)	0.061 (2)	−0.012 (3)	0.013 (2)	0.020 (2)
C18	0.0471 (16)	0.0411 (15)	0.0593 (18)	−0.0013 (12)	0.0149 (14)	0.0084 (13)
C19	0.083 (3)	0.0443 (17)	0.073 (2)	−0.0192 (17)	0.029 (2)	0.0007 (16)
C20	0.078 (3)	0.082 (3)	0.143 (5)	0.021 (3)	0.003 (3)	−0.027 (3)
C21	0.106 (4)	0.079 (3)	0.162 (6)	0.002 (3)	0.041 (4)	−0.036 (4)
C22	0.109 (4)	0.059 (3)	0.120 (4)	−0.021 (3)	0.033 (3)	−0.010 (3)
C23	0.112 (4)	0.080 (3)	0.129 (5)	0.000 (3)	−0.016 (4)	−0.027 (3)
C24	0.078 (3)	0.072 (3)	0.121 (4)	0.011 (2)	−0.009 (3)	−0.019 (3)
C25	0.080 (2)	0.0527 (19)	0.067 (2)	−0.0031 (17)	0.0260 (19)	0.0206 (16)
C26	0.086 (3)	0.091 (3)	0.095 (3)	−0.006 (2)	0.046 (3)	0.017 (3)

### Geometric parameters (Å, °)

**Table:**

Sn1—C1	2.128 (4)	C6'—C7'	1.551 (9)
Sn1—C5	2.134 (4)	C6'—H6'A	0.9700
Sn1—S3	2.5292 (9)	C6'—H6'B	0.9700
Sn1—S1	2.5425 (9)	C7'—C8'	1.504 (9)
Sn1—S4	2.8927 (9)	C7'—H7'A	0.9700
Sn1—S2	2.9257 (10)	C7'—H7'B	0.9700
S1—C9	1.744 (3)	C8'—H8'A	0.9600
S2—C9	1.691 (3)	C8'—H8'B	0.9600
S3—C18	1.743 (3)	C8'—H8'C	0.9600
S4—C18	1.693 (4)	C10—C11	1.494 (5)
N1—C9	1.329 (4)	C10—C15	1.516 (5)
N1—C16	1.472 (4)	C10—H10	0.9800
N1—C10	1.482 (4)	C11—C12	1.519 (5)
N2—C18	1.330 (4)	C11—H11A	0.9700
N2—C19	1.474 (5)	C11—H11B	0.9700
N2—C25	1.479 (5)	C12—C13	1.499 (6)
C1—C2	1.529 (8)	C12—H12A	0.9700
C1—C2'	1.530 (9)	C12—H12B	0.9700
C1—H1A	0.9700	C13—C14	1.496 (6)
C1—H1B	0.9700	C13—H13A	0.9700
C1—H1C	0.9700	C13—H13B	0.9700
C1—H1D	0.9700	C14—C15	1.526 (5)
C2—C3	1.527 (9)	C14—H14A	0.9700
C2—H2A	0.9700	C14—H14B	0.9700
C2—H2B	0.9700	C15—H15A	0.9700
C3—C4	1.530 (10)	C15—H15B	0.9700
C3—H3A	0.9700	C16—C17	1.512 (6)
C3—H3B	0.9700	C16—H16A	0.9700
C4—H4A	0.9600	C16—H16B	0.9700
C4—H4B	0.9600	C17—H17A	0.9600
C4—H4C	0.9600	C17—H17B	0.9600
C2'—C3'	1.526 (9)	C17—H17C	0.9600
C2'—H2'A	0.9700	C19—C20	1.481 (7)
C2'—H2'B	0.9700	C19—C24	1.510 (6)
C3'—C4'	1.531 (10)	C19—H19	0.9800
C3'—H3'A	0.9700	C20—C21	1.534 (8)
C3'—H3'B	0.9700	C20—H20A	0.9700
C4'—H4'A	0.9600	C20—H20B	0.9700
C4'—H4'B	0.9600	C21—C22	1.471 (8)
C4'—H4'C	0.9600	C21—H21A	0.9700
C5—C6	1.484 (9)	C21—H21B	0.9700
C5—C6'	1.505 (9)	C22—C23	1.459 (8)
C5—H5A	0.9700	C22—H22A	0.9700
C5—H5B	0.9700	C22—H22B	0.9700
C5—H5C	0.9700	C23—C24	1.521 (7)
C5—H5D	0.9700	C23—H23A	0.9700
C6—C7	1.567 (9)	C23—H23B	0.9700
C6—H6A	0.9700	C24—H24A	0.9700
C6—H6B	0.9700	C24—H24B	0.9700
C7—C8	1.491 (9)	C25—C26	1.493 (6)
C7—H7A	0.9700	C25—H25A	0.9700
C7—H7B	0.9700	C25—H25B	0.9700
C8—H8A	0.9600	C26—H26A	0.9600
C8—H8B	0.9600	C26—H26B	0.9600
C8—H8C	0.9600	C26—H26C	0.9600
			
C1—Sn1—C5	145.4 (2)	C8'—C7'—C6'	111.6 (10)
C1—Sn1—S3	102.1 (1)	C8'—C7'—H7'A	109.3
C5—Sn1—S3	102.5 (2)	C6'—C7'—H7'A	109.3
C1—Sn1—S1	103.7 (1)	C8'—C7'—H7'B	109.3
C5—Sn1—S1	102.1 (1)	C6'—C7'—H7'B	109.3
S3—Sn1—S1	85.37 (3)	H7'A—C7'—H7'B	108.0
C1—Sn1—S4	83.1 (1)	C7'—C8'—H8'A	109.5
C5—Sn1—S4	85.3 (1)	C7'—C8'—H8'B	109.5
S3—Sn1—S4	65.63 (3)	H8'A—C8'—H8'B	109.5
S1—Sn1—S4	151.00 (3)	C7'—C8'—H8'C	109.5
C1—Sn1—S2	84.4 (1)	H8'A—C8'—H8'C	109.5
C5—Sn1—S2	86.2 (2)	H8'B—C8'—H8'C	109.5
S3—Sn1—S2	150.15 (3)	N1—C9—S2	123.0 (2)
S1—Sn1—S2	64.83 (3)	N1—C9—S1	118.8 (2)
S4—Sn1—S2	144.13 (3)	S2—C9—S1	118.26 (18)
C9—S1—Sn1	94.1 (1)	N1—C10—C11	113.2 (3)
C9—S2—Sn1	82.6 (1)	N1—C10—C15	113.6 (3)
C18—S3—Sn1	93.3 (1)	C11—C10—C15	110.6 (3)
C18—S4—Sn1	82.4 (1)	N1—C10—H10	106.3
C9—N1—C16	121.2 (3)	C11—C10—H10	106.3
C9—N1—C10	120.7 (3)	C15—C10—H10	106.3
C16—N1—C10	118.2 (3)	C10—C11—C12	109.9 (3)
C18—N2—C19	121.2 (3)	C10—C11—H11A	109.7
C18—N2—C25	120.6 (3)	C12—C11—H11A	109.7
C19—N2—C25	118.2 (3)	C10—C11—H11B	109.7
C2—C1—Sn1	114.1 (11)	C12—C11—H11B	109.7
C2'—C1—Sn1	114.9 (11)	H11A—C11—H11B	108.2
C2—C1—H1A	108.7	C13—C12—C11	112.1 (4)
Sn1—C1—H1A	108.7	C13—C12—H12A	109.2
C2—C1—H1B	108.7	C11—C12—H12A	109.2
C2'—C1—H1B	117.4	C13—C12—H12B	109.2
Sn1—C1—H1B	108.7	C11—C12—H12B	109.2
H1A—C1—H1B	107.6	H12A—C12—H12B	107.9
C2—C1—H1C	118.2	C14—C13—C12	111.3 (3)
C2'—C1—H1C	108.5	C14—C13—H13A	109.4
Sn1—C1—H1C	108.5	C12—C13—H13A	109.4
C2'—C1—H1D	108.5	C14—C13—H13B	109.4
Sn1—C1—H1D	108.5	C12—C13—H13B	109.4
H1C—C1—H1D	107.5	H13A—C13—H13B	108.0
C3—C2—C1	110.4 (8)	C13—C14—C15	111.7 (3)
C3—C2—H2A	109.6	C13—C14—H14A	109.3
C1—C2—H2A	109.6	C15—C14—H14A	109.3
C3—C2—H2B	109.6	C13—C14—H14B	109.3
C1—C2—H2B	109.6	C15—C14—H14B	109.3
H2A—C2—H2B	108.1	H14A—C14—H14B	107.9
C2—C3—C4	110.1 (9)	C10—C15—C14	109.5 (3)
C2—C3—H3A	109.6	C10—C15—H15A	109.8
C4—C3—H3A	109.6	C14—C15—H15A	109.8
C2—C3—H3B	109.6	C10—C15—H15B	109.8
C4—C3—H3B	109.6	C14—C15—H15B	109.8
H3A—C3—H3B	108.2	H15A—C15—H15B	108.2
C3—C4—H4A	109.5	N1—C16—C17	114.3 (3)
C3—C4—H4B	109.5	N1—C16—H16A	108.7
H4A—C4—H4B	109.5	C17—C16—H16A	108.7
C3—C4—H4C	109.5	N1—C16—H16B	108.7
H4A—C4—H4C	109.5	C17—C16—H16B	108.7
H4B—C4—H4C	109.5	H16A—C16—H16B	107.6
C3'—C2'—C1	110.7 (8)	C16—C17—H17A	109.5
C3'—C2'—H2'A	109.5	C16—C17—H17B	109.5
C1—C2'—H2'A	109.5	H17A—C17—H17B	109.5
C3'—C2'—H2'B	109.5	C16—C17—H17C	109.5
C1—C2'—H2'B	109.5	H17A—C17—H17C	109.5
H2'A—C2'—H2'B	108.1	H17B—C17—H17C	109.5
C2'—C3'—C4'	110.1 (9)	N2—C18—S4	122.8 (3)
C2'—C3'—H3'A	109.6	N2—C18—S3	118.6 (3)
C4'—C3'—H3'A	109.6	S4—C18—S3	118.62 (18)
C2'—C3'—H3'B	109.6	N2—C19—C20	113.3 (4)
C4'—C3'—H3'B	109.6	N2—C19—C24	113.1 (4)
H3'A—C3'—H3'B	108.1	C20—C19—C24	110.3 (4)
C3'—C4'—H4'A	109.5	N2—C19—H19	106.5
C3'—C4'—H4'B	109.5	C20—C19—H19	106.5
H4'A—C4'—H4'B	109.5	C24—C19—H19	106.5
C3'—C4'—H4'C	109.5	C19—C20—C21	110.8 (5)
H4'A—C4'—H4'C	109.5	C19—C20—H20A	109.5
H4'B—C4'—H4'C	109.5	C21—C20—H20A	109.5
C6—C5—Sn1	121.0 (6)	C19—C20—H20B	109.5
C6'—C5—Sn1	109.5 (5)	C21—C20—H20B	109.5
C6—C5—H5A	107.1	H20A—C20—H20B	108.1
C6'—C5—H5A	105.6	C22—C21—C20	111.5 (5)
Sn1—C5—H5A	107.1	C22—C21—H21A	109.3
C6—C5—H5B	107.1	C20—C21—H21A	109.3
C6'—C5—H5B	120.2	C22—C21—H21B	109.3
Sn1—C5—H5B	107.1	C20—C21—H21B	109.3
H5A—C5—H5B	106.8	H21A—C21—H21B	108.0
C6—C5—H5C	109.8	C23—C22—C21	112.4 (5)
C6'—C5—H5C	109.8	C23—C22—H22A	109.1
Sn1—C5—H5C	109.8	C21—C22—H22A	109.1
C6'—C5—H5D	109.8	C23—C22—H22B	109.1
Sn1—C5—H5D	109.8	C21—C22—H22B	109.1
H5C—C5—H5D	108.2	H22A—C22—H22B	107.9
C5—C6—C7	108.7 (8)	C22—C23—C24	112.2 (5)
C5—C6—H6A	109.9	C22—C23—H23A	109.2
C7—C6—H6A	109.9	C24—C23—H23A	109.2
C5—C6—H6B	109.9	C22—C23—H23B	109.2
C7—C6—H6B	109.9	C24—C23—H23B	109.2
H6A—C6—H6B	108.3	H23A—C23—H23B	107.9
C8—C7—C6	108.9 (8)	C19—C24—C23	109.9 (5)
C8—C7—H7A	109.9	C19—C24—H24A	109.7
C6—C7—H7A	109.9	C23—C24—H24A	109.7
C8—C7—H7B	109.9	C19—C24—H24B	109.7
C6—C7—H7B	109.9	C23—C24—H24B	109.7
H7A—C7—H7B	108.3	H24A—C24—H24B	108.2
C7—C8—H8A	109.5	N2—C25—C26	114.8 (3)
C7—C8—H8B	109.5	N2—C25—H25A	108.6
H8A—C8—H8B	109.5	C26—C25—H25A	108.6
C7—C8—H8C	109.5	N2—C25—H25B	108.6
H8A—C8—H8C	109.5	C26—C25—H25B	108.6
H8B—C8—H8C	109.5	H25A—C25—H25B	107.5
C5—C6'—C7'	112.6 (9)	C25—C26—H26A	109.5
C5—C6'—H6'A	109.1	C25—C26—H26B	109.5
C7'—C6'—H6'A	109.1	H26A—C26—H26B	109.5
C5—C6'—H6'B	109.1	C25—C26—H26C	109.5
C7'—C6'—H6'B	109.1	H26A—C26—H26C	109.5
H6'A—C6'—H6'B	107.8	H26B—C26—H26C	109.5
			
C1—Sn1—S1—C9	74.03 (16)	C5—C6—C7—C8	−179.4 (18)
C5—Sn1—S1—C9	−82.86 (19)	C6—C5—C6'—C7'	−44 (8)
S3—Sn1—S1—C9	175.33 (12)	Sn1—C5—C6'—C7'	169.4 (12)
S4—Sn1—S1—C9	174.78 (12)	C5—C6'—C7'—C8'	82 (2)
S2—Sn1—S1—C9	−2.95 (12)	C16—N1—C9—S2	177.7 (3)
C1—Sn1—S2—C9	−104.97 (16)	C10—N1—C9—S2	−1.9 (5)
C5—Sn1—S2—C9	108.35 (19)	C16—N1—C9—S1	−2.3 (5)
S3—Sn1—S2—C9	−0.38 (16)	C10—N1—C9—S1	178.0 (2)
S1—Sn1—S2—C9	3.06 (12)	Sn1—S2—C9—N1	175.3 (3)
S4—Sn1—S2—C9	−175.06 (12)	Sn1—S2—C9—S1	−4.59 (18)
C1—Sn1—S3—C18	−76.12 (15)	Sn1—S1—C9—N1	−174.7 (3)
C5—Sn1—S3—C18	79.37 (17)	Sn1—S1—C9—S2	5.2 (2)
S1—Sn1—S3—C18	−179.20 (11)	C9—N1—C10—C11	105.0 (4)
S4—Sn1—S3—C18	0.51 (11)	C16—N1—C10—C11	−74.7 (4)
S2—Sn1—S3—C18	−176.08 (12)	C9—N1—C10—C15	−127.7 (4)
C1—Sn1—S4—C18	106.08 (15)	C16—N1—C10—C15	52.6 (4)
C5—Sn1—S4—C18	−106.54 (19)	N1—C10—C11—C12	−172.5 (4)
S3—Sn1—S4—C18	−0.52 (12)	C15—C10—C11—C12	58.7 (5)
S1—Sn1—S4—C18	0.08 (15)	C10—C11—C12—C13	−56.3 (6)
S2—Sn1—S4—C18	176.58 (12)	C11—C12—C13—C14	54.0 (6)
C5—Sn1—C1—C2	−8.3 (11)	C12—C13—C14—C15	−54.1 (6)
S3—Sn1—C1—C2	126.2 (10)	N1—C10—C15—C14	172.6 (3)
S1—Sn1—C1—C2	−145.7 (10)	C11—C10—C15—C14	−58.8 (5)
S4—Sn1—C1—C2	63.0 (10)	C13—C14—C15—C10	56.3 (5)
S2—Sn1—C1—C2	−83.3 (10)	C9—N1—C16—C17	−82.1 (4)
C5—Sn1—C1—C2'	4.1 (11)	C10—N1—C16—C17	97.6 (4)
S3—Sn1—C1—C2'	138.6 (10)	C19—N2—C18—S4	−0.9 (5)
S1—Sn1—C1—C2'	−133.3 (10)	C25—N2—C18—S4	179.4 (3)
S4—Sn1—C1—C2'	75.4 (10)	C19—N2—C18—S3	178.9 (3)
S2—Sn1—C1—C2'	−70.9 (10)	C25—N2—C18—S3	−0.8 (5)
C2'—C1—C2—C3	85 (11)	Sn1—S4—C18—N2	−179.4 (3)
Sn1—C1—C2—C3	−178.4 (15)	Sn1—S4—C18—S3	0.79 (17)
C1—C2—C3—C4	179 (2)	Sn1—S3—C18—N2	179.3 (3)
C2—C1—C2'—C3'	−67 (11)	Sn1—S3—C18—S4	−0.9 (2)
Sn1—C1—C2'—C3'	−155.8 (14)	C18—N2—C19—C20	−116.4 (5)
C1—C2'—C3'—C4'	−175 (2)	C25—N2—C19—C20	63.2 (5)
C1—Sn1—C5—C6	−4.2 (17)	C18—N2—C19—C24	117.0 (4)
S3—Sn1—C5—C6	−138.5 (17)	C25—N2—C19—C24	−63.3 (5)
S1—Sn1—C5—C6	133.6 (17)	N2—C19—C20—C21	175.2 (5)
S4—Sn1—C5—C6	−74.8 (17)	C24—C19—C20—C21	−56.8 (7)
S2—Sn1—C5—C6	70.3 (17)	C19—C20—C21—C22	55.0 (8)
C1—Sn1—C5—C6'	4.7 (14)	C20—C21—C22—C23	−53.6 (9)
S3—Sn1—C5—C6'	−129.6 (14)	C21—C22—C23—C24	54.7 (9)
S1—Sn1—C5—C6'	142.5 (14)	N2—C19—C24—C23	−174.8 (5)
S4—Sn1—C5—C6'	−65.9 (14)	C20—C19—C24—C23	57.0 (7)
S2—Sn1—C5—C6'	79.3 (14)	C22—C23—C24—C19	−55.9 (8)
C6'—C5—C6—C7	142 (11)	C18—N2—C25—C26	−82.2 (5)
Sn1—C5—C6—C7	179.4 (11)	C19—N2—C25—C26	98.1 (4)
